# Frontal Lobe Seizure Presenting as Disorganised Behaviour to a Mental Health Service: A Case Report

**DOI:** 10.1192/bjo.2025.10787

**Published:** 2025-06-20

**Authors:** Thilini Wickramarathna, Gayan Jayamaha, Miyuru Chandradasa, K.A.L.A. Kuruppuarachchi

**Affiliations:** 1 South West London and St George’s Mental Health NHS Trust, London, United Kingdom; 2 Victorian Institute of Forensic Mental Health, Victoria, Australia; 3 Faculty of Medicine, University of Kelaniya, Ragama, Sri Lanka

## Abstract

**Aims:** Epilepsy can present with a wide range of neuropsychiatric manifestations. A seizure episode may take the form of motor convulsions, complex abnormal behaviours or unusual subjective experiences.

Seizures originating in different anatomical locations take characteristic forms, however, there is considerable overlap in the presentation.

Frontal lobe seizures are characterised by motor phenomena which may include complex posturing and behavioural automatism which tend to begin and end abruptly.

This condition is of particular importance to psychiatrists, due to the bizarre nature of automatic behaviour. They may be mistaken as dissociative phenomena or psychotic disorders.

**Methods:** A 55-year-old female who was previously well, presented to the psychiatric service following episodic disorganised behaviour for two weeks duration. For example, she had cooked rice three times per meal without an apparent reason. Also, she had started collecting a large number of vegetables repeatedly in a shopping centre without a clear purpose. At that time her husband had to forcefully stop her. When inquired later she was unable to recall these events. Family members also noticed that the patient is having episodic repetitive facial movements without losing consciousness.

General physical and neurological examination was normal. Her EEG showed right frontal sharp waves which progress into generalised spikes, suggestive of frontal lobe epilepsy with secondary generalisation. The contrast CT brain was normal. Haematological tests including random blood sugar, serum electrolytes, full blood count, serum calcium levels and the ECG were normal. Intravenous phenytoin sodium was given to control repetitive seizures at the onset.

Subsequently, oral sodium valproate was commenced. She responded well, and symptoms disappeared within a couple of days.

**Results:** Epilepsy presenting as behavioural and psychiatric manifestations is common but can be easily overlooked. Frontal lobe epilepsy with common aetiology like post traumatic, tumours and genetic causes can have complex seizure semiology.

Overall frontal lobe seizures tend to begin and end abruptly, are brief and frequent. They show a tendency to occur at night and in clusters. Motor phenomena which may include complex posturing and behavioural automatism are usually the most conspicuous feature.

This patient had several bizarre behaviours which could be complex behavioural automatism. The bizarre nature of automatism means that they can often be mistaken for non-epileptic dissociative seizures as well as another psychiatric diagnosis like mania or psychosis.

**Conclusion:** Our patient highlights the unusual way of presentation of frontal lobe seizures. Clinicians need to be aware of this presentation to minimise possible misdiagnosis and mismanagement.

